# Fosfomycin Uptake in *Escherichia coli* Is Mediated by the Outer-Membrane Porins OmpF, OmpC, and LamB

**DOI:** 10.1021/acsinfecdis.3c00367

**Published:** 2023-12-17

**Authors:** Martina Bianchi, Mathias Winterhalter, Theresa Anisja Harbig, Daniel Hörömpöli, Ishan Ghai, Kay Nieselt, Heike Brötz-Oesterhelt, Christoph Mayer, Marina Borisova-Mayer

**Affiliations:** † Department of Organismic Interactions, Interfaculty Institute of Microbiology and Infection Medicine (IMIT), University of Tübingen, 72076 Tübingen, Germany; ‡ Department of Life Sciences and Chemistry, Constructor University, 28759 Bremen, Germany; § Institute for Bioinformatics and Medical Informatics, University of Tübingen, 72076 Tübingen, Germany; ∥ Department of Microbial Bioactive Compounds, IMIT, University of Tübingen, 72076 Tübingen, Germany; ⊥ Cluster of Excellence “Controlling Microbes to Fight Infections” University of Tübingen, 72076 Tübingen, Germany

**Keywords:** fosfomycin, Escherichia coli, outer-membrane porin, OmpF, OmpC, LamB

## Abstract

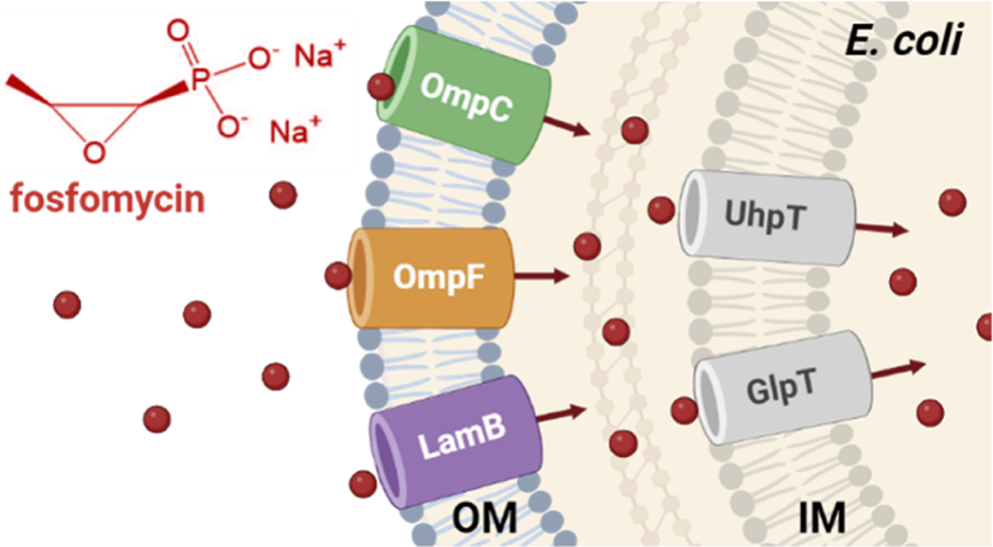

The antibiotic fosfomycin (FOS) is widely recognized for the treatment of lower urinary tract infections with *Escherichia coli* and has lately gained importance as a therapeutic option to combat multidrug-resistant bacteria. However, resistance to FOS frequently develops through mutations reducing its uptake. Although the inner-membrane transport of FOS has been extensively studied in *E. coli*, its outer-membrane (OM) transport remains insufficiently understood. While evaluating minimal inhibitory concentrations in OM porin-deficient mutants, we observed that the *E. coli* Δ*ompF*Δ*ompC* strain is four times more resistant to FOS than the wild type and the respective single mutants. Continuous monitoring of FOS-induced lysis of porin-deficient strains additionally highlighted the importance of LamB. The relevance of OmpF, OmpC, and LamB to FOS uptake was confirmed by electrophysiological and transcriptional analysis. Our study gives for the first time in-depth insight into the transport of FOS through the OM in *E. coli*.

The bacterial cell envelope of Gram-negative “diderm” bacteria consists of an inner membrane (IM), a cell wall peptidoglycan layer, and an outer membrane (OM).^1^ The OM functions as a robust permeability barrier and comprises a unique asymmetric bilayer, with an inner leaflet of two-tailed phospholipids and an outer leaflet of lipopolysaccharides.^2^ The latter restricts the passage of hydrophobic compounds into the cell, whereas hydrophilic molecules, including antibiotics or nutrient factors, can passively diffuse through specific water-filled channels named porins.^3−7^ In *Escherichia coli*, the two major OM porins OmpF and OmpC possess 16-stranded β-barrel structures^8,6,9^ and a pore size that allows the translocation of charged molecules up to 600 Da.^10^ OmpC and OmpF preferentially transport small cationic molecules but are mostly nonspecific channels,^11−14^ and they occur in the OM either as homotrimers of three identical subunits^8,9^ or as heterotrimers of mixed OmpF and OmpC subunits.^15^ PhoE is an inducible porin in *E. coli*, which is derepressed under phosphate limitation,^16^ and it preferentially facilitates the diffusion of anionic molecules.^11−13,17^ PhoE shares around 60% overall amino acid similarity with OmpF and OmpC and can form homotrimers or heterotrimers with the latter two porins.^15^ Another inducible general porin of *E. coli* is LamB, which is more distantly related to OmpF, OmpC and PhoE. It specifically uptakes the sugars maltose and α-1,4-linked maltodextrins up to maltoheptaose,^18−20^ and its expression is induced by intracellular maltotriose.^21^

Prolonged antibiotic treatment was shown to induce a loss of porin protein expression or the emergence of specific point mutations in genes encoding OM channels in clinical isolates of Gram-negative pathogens.^7^ Despite the occurence of porin-mediated antibiotic resistances, a profound and systematic investigation of the outer-membrane channels controlling the passage of clinically relevant drugs is still lacking.^22^ For example, there is poor knowledge about the OM permeation of fosfomycin (FOS), an “old” drug whose clinical potential has recently been re-evaluated in light of the current antibiotic resistance crisis.

FOS is a broad-spectrum bactericidal antibiotic that acts intracellularly as a phosphoenolpyruvate analogue. It inhibits the first committed step of bacterial peptidoglycan synthesis, by covalently binding to the Cys_115_ residue (*E. coli* numbering) of the UDP-*N*-acetylglucosamine-3-enolpyruvyl transferase enzyme MurA.^23−25^ From a chemical point of view, FOS is a small (138 Da) anionic molecule containing an epoxide ring and a phosphonate group^23,26^ and it is biologically active at physiological pH in its monoanionic form^27^ (predicted p*K*_a_ values of 1.25 and 7.82, as shown in https://chemicalize.com/#/calculation). Its oral formulation, FOS Trometamol (i.e., TRIS-buffered FOS), is routinely applied for the treatment of urinary tract infections.^28,29^*In vitro* and *in vivo* studies, as well as clinical trials, revealed the efficacy of this drug against multidrug-resistant and even biofilm-forming bacteria.^30,31,29^ Therefore, the intravenous formulation of FOS disodium salt is currently recommended for the treatment of pulmonary exacerbations in cystic fibrosis patients.^32^ Since FOS has a unique chemical structure and mechanism of action, as well as a good tissue distribution and favorable safety profile,^26,33,34^ it is also successfully used for the treatment of multidrug-resistant bacteria in a combinatorial therapy with other classes of antibiotics.^35,36^

The active uptake of FOS through the inner membrane of *E. coli* has been extensively studied and is carried out by two sugar antiporters, the hexose phosphate transporter (UhpT) and the glycerol 3-phosphate transferase system (GlpT). The transporters UhpT and GlpT are induced by extracellular glucose 6-phosphate (G6P) and intracellular glycerol 3-phosphate (G3P), respectively.^24,37−40^ Supplementation of 25 μg/mL G6P is recommended by EUCAST when testing the susceptibility of *E. coli* to FOS, to ensure UhpT expression and FOS uptake.^24,41^ Interestingly, agar dilution test results showed in *E. coli* BW25113 strains that the minimal inhibitory concentration (MIC) for FOS increased 32-fold in the Δ*uhpT* mutant (64 μg/mL) relative to the wt (2 μg/mL), whereas the MIC values of the Δ*glpT* and the parental strain were identical.^42^ This suggests that UhpT is the preferred route for FOS entrance under standard conditions in this *E. coli* strain.

While FOS transport through the IM has been well investigated, its passage through the OM remains enigmatic. So far, electrophysiology experiments combined with all-atom dynamic simulations showed the translocation of FOS through OmpF.^43^ The same year, however, a study by Choi *et al*. suggested no role of OmpF, OmpC, and LamB in FOS uptake, by measuring identical MICs in the *E. coli* MG1655 strain and respective porin deletion mutants.^44^ However, these agar dilution experiments were performed in the absence of G6P and, thus, without induction of the inner-membrane transporter for FOS UhpT, which may have biased their results.^44^

We argue that under conditions of rapid translocation across the IM, the transport through the OM may become rate-limiting, thereby explaining the contradictory results regarding the role of OM porins in FOS uptake. To better understand porin-mediated resistance to FOS in *E. coli* and to evaluate the critical role of UhpT for the OM porin-mediated uptake of FOS, we conducted whole-cell experiments and compared the susceptibility of a BW25113 wild type (wt) strain and the corresponding porin-deficient *ompF*, *ompC*, and *lamB* mutants with and without induction of UhpT expression. We further assessed the physiological role of OmpF, OmpC, and LamB by evaluating their transcript abundance in the cells and by estimating electrophysiologically the flux of FOS through the different trimeric porin channels. Our findings indicate a crucial role for OmpF, OmpC, and LamB in the uptake of FOS under UhpT-inducing conditions.

## Results

### Role of OmpF and OmpC in FOS Susceptibility Revealed under UhpT-Inducing Conditions

**Figure 1:**
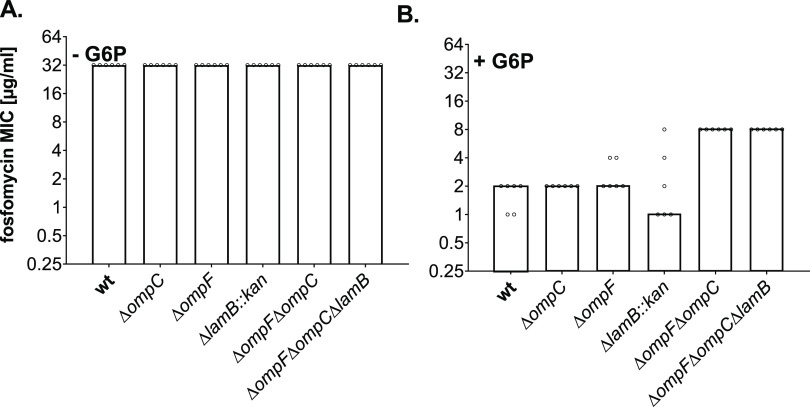
FOS susceptibility of *E. coli* wild type and porin mutant strains determined by the agar dilution method. Overnight cultures of *E. coli* BW25113 wild type (wt), Δ*ompC*, Δ*ompF*, and Δ*lamB::kan* single mutants, as well as Δ*ompF*Δ*ompC* double and Δ*ompF*Δ*ompC*Δ*lamB* triple mutants were diluted in 0.85% NaCl and 2 × 10^4^ cfu were spotted on LB agar plates without (control) or with 0.25–512 μg/mL two times serial dilutions of FOS (A) in the absence (−G6P) or (B) in the presence of 25 μg/mL G6P (+G6P). The MICs are shown as mode values [μg/mL] from three biological replicates, each done in technical duplicates. The *y*-axis is presented on a log_2_ scale.

We aimed to revisit the physiological role of OmpF, OmpC, and LamB in the uptake of FOS, first repeating the experiments of Choi and Lee,^44^ but also including UhpT-inducing conditions. We reproduced the agar dilution method on lysogeny broth (LB) agar plates in the absence and in the presence of 25 μg/mL G6P and compared the MIC values for FOS of the *E. coli* BW25113 parental strain (wt), the single mutants Δ*ompC*, Δ*ompF* and Δ*lamB::kan*, as well as a double Δ*ompF*Δ*ompC* and a triple Δ*ompF*Δ*ompC*Δ*lamB* mutant. Correct gene deletions in these strains were verified by colony PCR as shown in Figure S1. In line with the previous observations,^44^ the wt and the porin mutants grown in the absence of G6P showed no differences in their FOS sensitivity, as all tested strains had a MIC value of 32 μg/mL (Figures 1A and S2A). However, when the LB agar plates were supplemented with G6P, we observed differences in the susceptibility between the strains (Figures 1B and S2B). The MIC value of the wt decreased dramatically by 16 times, from 32 to 2 μg/mL, likely due to the expression of the UhpT transporter, and also the MIC values of the single mutants Δ*ompC*, Δ*ompF*, and Δ*lamB::kan* dropped to 2, 2, and 1 μg/mL, respectively, thus indicating similar susceptibility in comparison to the wt. Intriguingly, the Δ*ompF*Δ*ompC* double mutant became 4 times more resistant to FOS compared to the parental strain in the presence of G6P, with a MIC value of 8 μg/mL (Figures 1B and S2B). Our data reveal the role of the porins OmpF and OmpC in the FOS susceptibility, which became detectable only when UhpT was induced. We did not note differences in the MIC values when comparing the Δ*ompF*Δ*ompC* and Δ*ompF*Δ*ompC*Δ*lamB* strains, indicating that the maltoporin LamB has no major role under the tested experimental conditions (Figures 1 and S2).

### Gradient Strip Test Confirms the Role of OmpF and OmpC in FOS Susceptibility

**Figure 2:**
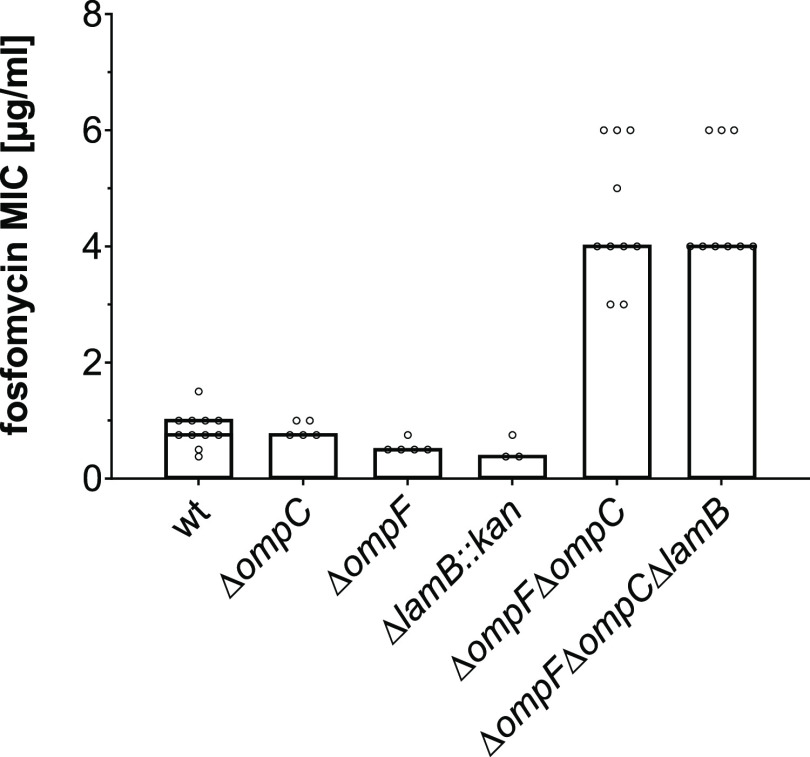
Susceptibility of *E. coli* wt and porin mutant strains to FOS by gradient strip test. Overnight cultures of *E. coli* wt, Δ*ompC*, Δ*ompF*, Δ*lamB::kan*, as well as Δ*ompF*Δ*ompC* and Δ*ompF*Δ*ompC*Δ*lamB* mutants were diluted in 0.85% NaCl to McFarland 0.5. Susceptibility was assayed on LB agar plates with MIC Test Strips (MTS) for FOS (0.064–1024 μg/mL), which contain 50 μg/mL G6P. The MICs [μg/mL] from at least three biological replicates are presented as mode values. For *E. coli* wt strain, we obtained two mode values of 0.75 and 1 μg/mL.

We further studied the role of the OmpF, OmpC, and LamB porins by gradient strip test, using commercially available high FOS MIC Test Strips (MTS) (0.064–1024 μg/mL) that contain 50 μg/mL G6P for induction of UhpT expression. We evaluated the MIC following Liofilchem’s recommendations and ignored the emergence of isolated resistant colonies within the zone of inhibition (Figure S3A) since high rates of spontaneous mutations occur when FOS susceptibility is tested in *E. coliin vitro*.^42^ Similar to the agar dilution method, no increase in the MIC values was observed when comparing *E. coli* wt (0.75–1 μg/mL) and the respective single-porin mutants, lacking *ompC* (0.75 μg/mL), *ompF* (0.5 μg/mL) and *lamB* (0.38 μg/mL) genes (Figure 2). Once again, the double Δ*ompF*Δ*ompC* porin mutant showed a MIC of 4 μg/mL, which indicates that this strain is ≥4 times more resistant to FOS, when compared to the wt. A MIC of 4 μg/mL was also determined for the Δ*ompF*Δ*ompC*Δ*lamB* mutant (Figures 2 and S3A). The importance of OmpF and OmpC in uptake was recently shown for other antibiotics like meropenem,^45^ which we confirmed by gradient strip test (Figure S3B).

### Additional Role of LamB in FOS Susceptibility Revealed in Liquid Medium

**Figure 3:**
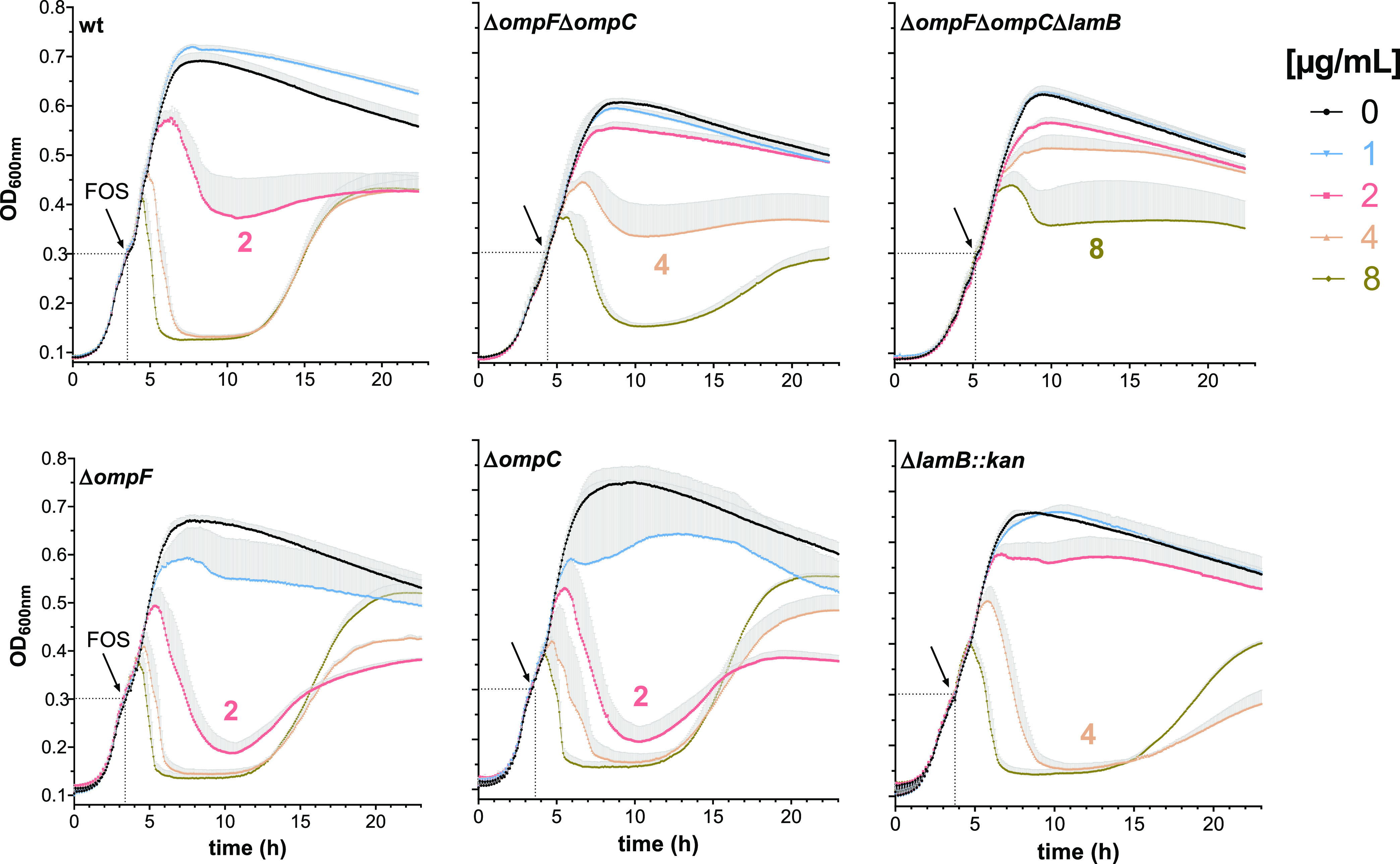
Continuous monitoring of *E. coli* wt and porin mutant cell lysis and regrowth in liquid LB prior and after addition of FOS. The OD_600_ of *E. coli* wt, Δ*ompC*, Δ*ompF*, and Δ*lamB::kan*, Δ*ompF*Δ*ompC* and Δ*ompF*Δ*ompC*Δ*lamB* porin mutants overnight cultures was adjusted in LB medium with G6P to 0.05. Cell growth was monitored every 10 min in the microplate reader, and when an OD_600_ of 1.9 (value measured in a cuvette, corresponding to 0.3 in the plate reader) was reached, FOS (final concentrations of 1–8 μg/mL) or water (0 μg/mL FOS) was added to the cells. Arrows indicate the time of the FOS addition. Growth curves are shown as the mean of 3–5 biological replicates + standard error of the mean (SEM).

Experiments performed on agar plates enable determination of the final MIC for FOS as an end-point read-out but disregard differences in the susceptibility shortly after exposure to the drug. Therefore, to determine whether the wt and the OmpF, OmpC, and LamB deficient mutants differ in their susceptibility to FOS when exposed to the drug at mid-exponential growth phase, we compared the lysis and regrowth process in liquid culture (Figure 3). We observed that the wt and the Δ*ompC* and Δ*ompF* mutants had a similar FOS sensitivity, lysing with 2 μg/mL FOS, whereas the Δ*lamB::kan* mutant was more resistant, lysing with 4 μg/mL. The Δ*ompF*Δ*ompC* and Δ*ompF*Δ*ompC*Δ*lamB* mutants were also more resistant than the wt strain and lysed with 4 μg/mL and 8 μg/mL, respectively (Figure 3). These results confirm the role of OmpF and OmpC in FOS susceptibility and reveal an additional contribution of LamB to the liquid LB medium.

To confirm the role of LamB, we additionally compared the FOS susceptibility of all strains by a gradient strip test on LB agar plates with and without maltotriose supplementation (Figure S4). Maltoriose is the inducer of the maltose regulon, to which *lamB* also belongs.^21^ As expected upon LamB expressing conditions, the MIC of the Δ*ompF*Δ*ompC* strain decreased by one-third from 3 μg/mL (−maltotriose) to 2 μg/mL (+maltoriose), while no decrease in the FOS sensitivity was observed for the Δ*ompF*Δ*ompC*Δ*lamB* strain. The effect of LamB induction was also visible in the wt strain with a lower magnitude (Figure S4).

### Wt and Porin-Deficient Strains Have Similar *uhpT* Expression Levels at the Time of FOS Administration

**Figure 4:**
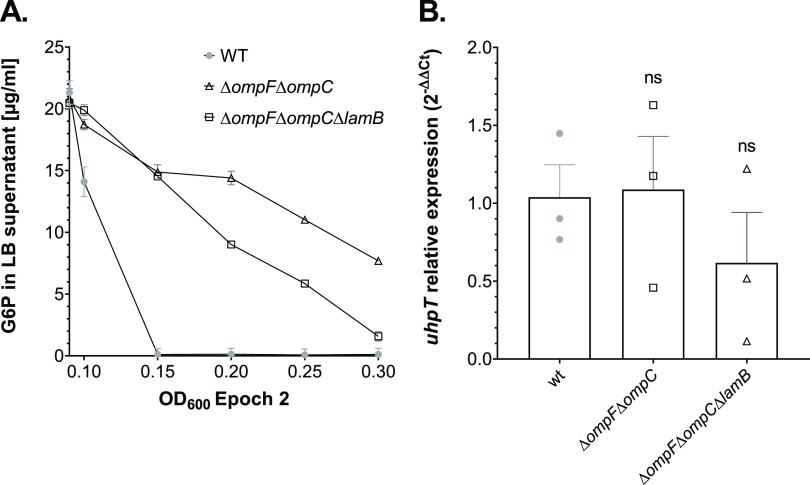
G6P quantification in the LB culture medium and relative *uhpT* expression of wt and porin-deficient strains. Overnight cultures of *E. coli* wt, Δ*ompF*Δ*ompC*, and Δ*ompF*Δ*ompC*Δ*lamB* porin mutants were diluted in LB medium with G6P (25 μg/mL) to a final OD_600_ of 0.05. Cell growth was monitored in the microplate reader until an OD_600_ of 1.9 (0.3 in the Epoch 2 plate reader) was reached. (A) Detection of G6P amounts in the culture medium of *E. coli* wt and porin-deficient strains was performed by HPLC-MS. Culture supernatants were collected at time point 0 (OD_600_ of 0.09 in the plate reader) and at every OD_600_ increase of 0.05 up to an OD_600_ value of 0.3. Extracted ion chromatograms (EICs) for G6P (observed mass [M – H]^−^ of 259.022 *m*/*z* and maximal peak intensity at 17 min) and the area under the curve (AUC) were calculated with the UmetaFlow program. The concentration of G6P in each sample was calculated by using a standard curve for this metabolite (Figure S5). The experiment was performed in biological duplicates, and the G6P values were presented as mean ± SEM. (B) Relative quantification of *uhpT* mRNA expression was performed by reverse transcription-quantitative polymerase chain reaction (RT-qPCR). 16S rRNA was used as a housekeeping gene for internal standardization. Data are presented as *uhpT* relative expression (2^–ΔΔCt^) as mean ± SEM of three biological replicates, each done in two technical duplicates. Statistical analysis with one-way analysis of variance (ANOVA) with Dunnett’s multiple comparison test in comparison to wt strain (ns, not significant).

It is likely that the increased FOS resistance in the Δ*ompF*Δ*ompC* and Δ*ompF*Δ*ompC*Δ*lamB* mutants is due to a decreased drug uptake by the OmpF, OmpC, and LamB porins. Still, we cannot exclude that their deletion also has an indirect effect, causing lower G6P permeation through the OM, and thereby lower UhpT expression on the IM. We therefore quantified the amount of residual G6P in the supernatant of the *E. coli* wt and the porin-depleted strains grown in LB medium with 25 μg/mL G6P by high-performance liquid chromatography coupled to mass spectrometry (HPLC-MS) using a standard G6P curve (Figures 4A and S5A). The LB medium was deficient for this metabolite; however, we detected another compound with an identical mass as G6P, possibly another C6 phosphate, eluting on the HILIC column with the same retention time of 17 min (Figure S5B). Interestingly, we observed that the wt strain had consumed the 25 μg/mL G6P completely while grown to an OD_600_ of 0.15 (Figures 4A and S5C) and at an OD_600_ between 0.15 and 0.3 the supernatant retained the compound with the identical mass as G6P, which could not be internalized by the wt cells (Figure S5C). In comparison, the Δ*ompF*Δ*ompC* and the Δ*ompF*Δ*ompC*Δ*lamB* cells utilized the G6P from the LB medium slower, as residual amounts of 30 and 6.3%, respectively, were still detected at the time of FOS supplementation (OD_600_ of 0.3) (Figures 4A and S5C). Even if the consumption of the G6P in the absence of the porins OmpC, OmpF, and LamB was delayed (Figure 4A), no significant difference in the *uhpT* mRNA expression was observed between the wt and the porin-deficient strains (Figure 4B). These results suggest that FOS internalizes through UhpT with similar rates in all investigated strains at the time of drug supplementation.

### Physiological Relevance of OmpF, OmpC, and LamB for Translocation of FOS

Previously, electrophysiological studies have shown that OmpF is able to transport FOS at a high rate.^43^ Here, we extend the study to other highly abundant porins like OmpC or LamB. We also included PhoE in our experiments, which shares 60% similarity to OmpF and OmpC, and preferentially translocates small anionic molecules and thus potentially also the negatively charged FOS.^13^ As performed by Golla *et al*., we reconstituted the respective purified recombinant porins into planar lipid membranes and measured the ion current and the reversal potential, as summarized in Table 1. We first measured the single-channel conductance using 25 mM FOS disodium salt (FOSNa_2_) or NaCl on both sides of the lipid bilayer (Table 1). Note that the two larger cation-selective channels, OmpF and OmpC, showed an increase in conductance in the case of FOSNa_2_, indicating that it is almost fully dissociated leading to a higher Na^+^ concentration. In the case of PhoE, Na^+^ ions contribute less to the conductance as it is an anion-selective channel. In contrast, LamB has an intrinsic low conductance which is mainly determined by the ionized amino acids inside the small channel. As expected, LamB showed almost no change in the conductance. In addition, we measured the reversal potential by increasing the concentration of FOSNa_2_ or NaCl to 90 mM on the ground side of the lipid bilayer (Table 1). The reversal potential measured for all porins applying the drug or NaCl had a value below 32 mV (detected only if both counterions of one molecule are able to permeate), which indicates that FOS is indeed translocated by all four channels. PhoE as an anion-selective channel gives a negative reversal potential in the case of a NaCl gradient. In contrast, in the presence of a FOSNa_2_ gradient, the positive potential indicated a relatively faster permeation of Na compared to FOS. This could be due to the smaller size of Na^+^ in comparison to FOS. Another explanation could be an affinity of FOS to the channel surface, leading to a deceleration of the drug anion permeation. The flux of FOS molecules linearly extrapolated to 1 μM of the drug for the porins OmpF, PhoE, OmpC, and LamB was, respectively, 37,^43^ 9, 7, and 3 molecules/s and per monomer (Table 1).

**Table 1:** Translocation of FOS through the Porins OmpF, OmpC, LamB, and PhoE^a^

	single-channel conductance (pS)		reversal potential (mV)		
porin	FOSNa_2_	NaCl	FOSNa_2_	NaCl	flux extrapolated to 1 μM (FOS molecules/s)
OmpF	88 ± 6	59 ± 15	11.2 ± 1.9	14.1 ± 2.3	37
OmpC	75 ± 5	37 ± 10	22 ± 3	8.9 ± 2.8	7
PhoE	76 ± 2	96 ± 13	12.7 ± 8	–11.2 ± 3	9
LamB	6.5 ± 2	6.2 ± 2	9.7 ± 3	11 ± 8	3

a The analysis of single trimeric channel conductance for the respective porins was performed by adding 25 mM FOS disodium salt (FOSNa_2_) on both sides of the lipid bilayer. For comparison, we show the corresponding values for 25 mM NaCl. In the next step, the salt concentration (NaCl or FOSNa_2_) at the electrical ground side of the amplifier was increased to 90 mM, and the reversal potential was measured. The estimated number of FOS molecules permeating across the channel was determined by extrapolating to a concentration gradient of 1 μM FOS, as previously shown.^43^ Notably, the sensitivity of the electrodes does not allow μM gradients, and thus the translocation numbers are rather order of magnitude estimates. Moreover, the underlying theory neglects a potential interaction between the ions and the bacterial cell wall and is based on assuming a homogeneous gradient across the channel. All measurements were recorded 10 times at 20 °C in 4-(2-hydroxyethyl)-1-piperazineethanesulfonic acid (HEPES) buffer at pH 7. Translocation data for FOS by OmpF are from Golla *et al*.^43^

**Figure 5:**
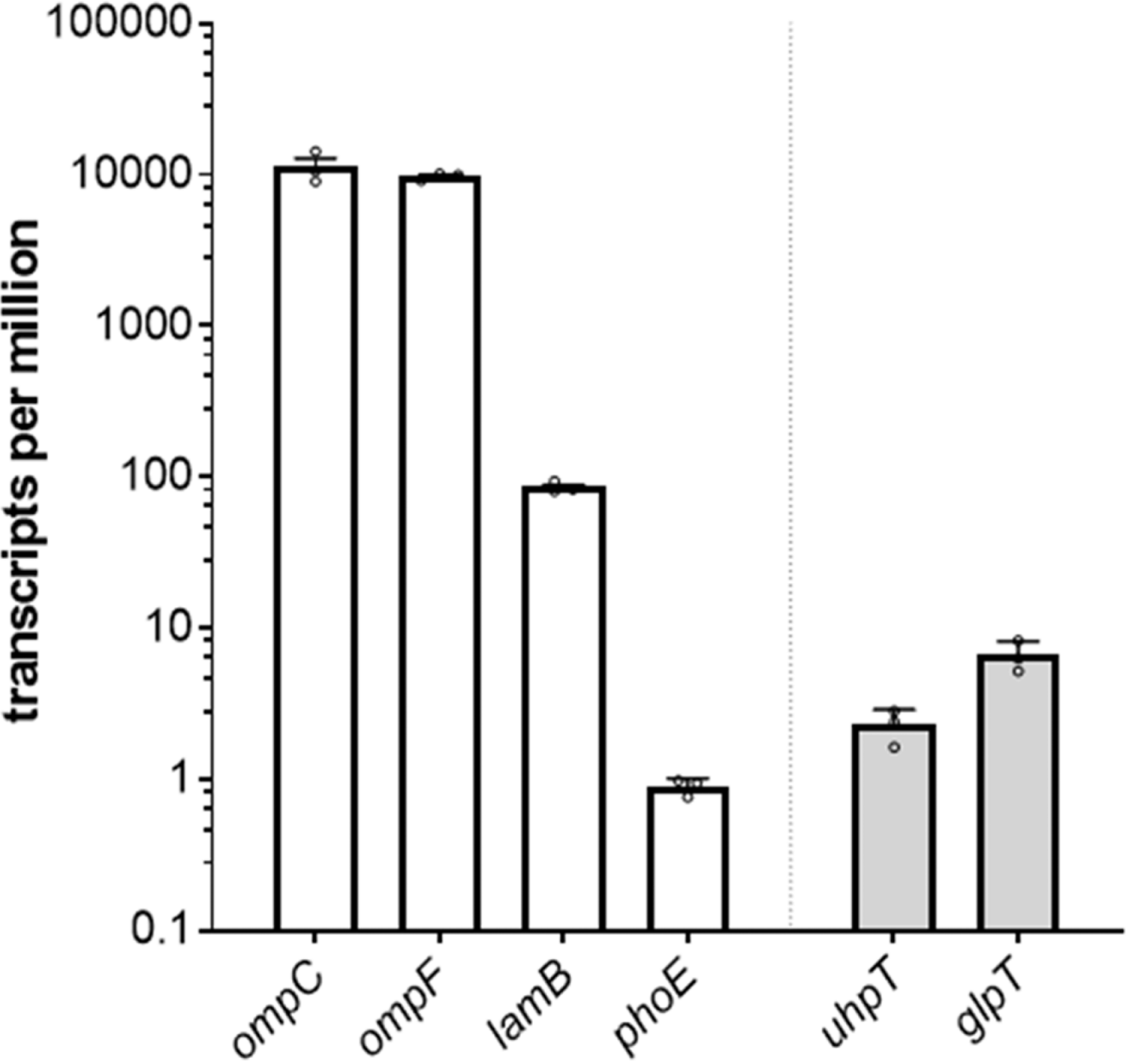
Transcripts per million presented for *E. coli* BW25113 genes encoding porins and the inner-membrane transporters for FOS UhpT and GlpT. *E. coli* BW25113 cells were grown in LB medium with an OD_600_ of 0.3 in the microplate reader, corresponding to an OD_600_ of 1.9 in the cuvette. Cells were harvested, total RNA was extracted, and after Illumina RNA sequencing, total reads were mapped and normalized as transcripts per million (TPM) as described in the Methods section and presented as transcripts per million (TPM) on a log-10 y-scale. The experiment was performed in three biological replicates, and data were presented as mean + SEM on a log-10 y-scale.

Since all of the tested porins are able to translocate the drug, we asked ourselves which porin has the largest physiological relevance under our growth conditions (LB medium at OD 1.9 in the cuvette, corresponding to 0.3 in the plate reader). To estimate their abundance, we compared their respective expression levels by Illumina RNA sequencing. For each of these four porins, we computed the number of transcripts per million (TPM) and compared them to the TPM values obtained for the inner-membrane transporters UhpT and GlpT (Figure 5). This transcriptional analysis allowed us to calculate the exact number of transcripts under our growth conditions in LB medium at the time of FOS exposure at an OD_600_ = 1.9. Our RNA-seq results showed that under standard growth conditions, both OmpF and OmpC are indeed very highly expressed porins in the cell with 11,261 and 9737 transcripts per million (TPM), respectively. LamB occurred with 85 TPM and the lowest expression was detected for PhoE with only 0.9 TPM (Figure 5). Therefore, we consider the latter to be not physiologically relevant for the transport of FOS in our setup. Maybe more interesting was the finding that the TPM counts for GlpT and for UhpT of 6.6 and 2.3, respectively, were much lower, compared to those obtained for the porins OmpF, OmpC, and LamB (Figure 5), indicating that the IM transporters are only moderately expressed. A list of the TPM values for so far known porins of *E. coli* is provided in Figure S6.

## Discussion

As antibiotic resistance caused by Gram-negative bacteria gained increased attention as a global health problem, there is an urgent demand to understand how drugs internalize through the outer membrane.^22^ In a previous electrophysiological *in vitro* study recombinantly purified OmpF was shown to translocate FOS.^43^ Our study combines *in vivo* and *in vitro* experiments, such as whole-cell susceptibility assays, growth monitoring, metabolite assays, and electrophysiological measurements, as well as transcriptional analysis of porin abundance to investigate the role of the outer-membrane porins OmpF, OmpC and LamB for the import of FOS into *E. coli* cells.

Previous agar dilution experiments with wt and porin-deficient strains conducted by Choi and Lee showed no role of OmpF, OmpC, and LamB for FOS internalization.^44^ However, this group did not consider in their experimental setup the importance of UhpT expression, which is only sufficiently induced for FOS uptake across the inner membrane by supplementation of the medium with 25 μg/mL glucose 6-phosphate (G6P), a procedure recommended specifically for this drug by EUCAST.^41^ In the absence of G6P, our results were in agreement with the report of Choi and Lee, as we obtained a high MIC of 32 μg/mL for all strains (Figure 1A). However, in the presence of G6P and consequently upon *uhpT* induction, not only were all strains more sensitive to FOS (Figure 1B), but also the *E. coli* Δ*ompF*Δ*ompC* mutant turned out as much as 4 times more resistant than the wt (Figure 1B). As the MICs of the single-porin mutants were comparable to the MIC of the wt, we conclude that both major porins, OmpF and OmpC, are relevant for the translocation of FOS into *E. coli* cells, each of them substituting for the loss of the other (Figures 1 and 2). Consistently, OmpF and OmpC-mediated resistances in *E. coli* were previously shown for different classes of β-lactams such as carbapenems, penicillins, cephalosporins,^22,44,46−49^ as well as fluoroquinolones,^50^ colicin^51^ and kanamycin.^45,52^ In addition, OmpF-related resistance alone was reported for the antibiotics tetracycline, aztreonam, cinoxacin, and clindamycin,^44^ and OmpC was related to an increased resistance to trimethoprim,^53^ tetracycline^54^ and cefepime.^55^

Besides confirming the prevalent role of OmpF and for the first time demonstrating a contribution of OmpC in FOS translocation, we further revealed the role of LamB in the uptake of FOS by continuous turbidimetric monitoring of *E. coli* growth in liquid LB medium and by gradient strip test on LB agar plates supplemented with 100 μM maltotriose (Figures 3 and S4). LamB-mediated internalization of FOS solely seen in liquid LB medium could be explained by the lower level of porin expression, as the number of mRNA transcripts for OmpF and OmpC was 100 times higher than the transcript levels detected for LamB (Figure 5). In previous reports, down-regulation or deletion of LamB was linked to tetracycline resistance^56,54^ and decreased expression of the LamB/pyruvate dehydrogenase E1 component (AceE) complex was detected in clinical *E. coli* strains resistant to ciprofloxacin, chlortetracycline, nalidixic acid, balofloxacin and ceftriaxone sodium.^57^

We showed the transport of FOS by the porins also by electrophysiological studies, finding that the FOS flux was highest through OmpF,^43^ followed by OmpC and LamB (Table 1). We also tested the channel permeability for the porin PhoE, showing FOS flux similar to that for the porins OmpC and LamB (Table 1). However, we do not consider this porin as physiologically relevant in phosphate-rich LB medium, as only low amounts of mRNA transcripts of 0.9 TPM were detected for PhoE (Figure 5) in the *E. coli* cells at the time of the drug treatment. Still, it would be interesting to investigate the role of PhoE for the FOS uptake under phosphate-limiting conditions, under which this porin is highly induced.^16^ To our knowledge, to date *in vitro* or *in vivo* studies revealing the role of PhoE for the uptake of antibiotics are lacking. However, electrophysiological analysis revealed that the OprO and OprP porins of *Pseudomonas aeruginosa*, which transport pyrophosphate and phosphate molecules, respectively, are able to internalize FOS at much lower rates.^58,59^

Even if mRNA levels may not exactly reflect protein amounts within the cell, our transcriptional data could be of broader interest for the community as they include the expression levels of all *E. coli* BW25113 genes in the routinely used culture medium LB (NCBI GEO accession number GSE236554). We compared the TPM of the so far known porins of *E. coli* (Figure S6). As FOS is a very small molecule, we expected that other OM channels could be relevant for its transport, even when transcribed in few copies. Indeed, ChiP (2.5 TPM in LB medium, Figure S6) was shown to still be sufficient to internalize the aminoglycosides kanamycin, gentamycin, and amikacin.^52^ Another intriguing result was that the transcripts for UhpT and GlpT inner-membrane transporters are much less abundant in comparison to the transcripts for OmpF, OmpC, and LamB (Figure 5), suggesting that the IM, rather than the OM might be the rate-limiting barrier for FOS uptake in LB. In agreement, we showed the role of the OmpF, OmpC, and LamB porins for the uptake of the drug only upon UhpT-inducing conditions (Figures 1–3).

## Conclusions

The translocation of antibiotics across the OM of Gram-negative bacteria via porins and the contribution of porins to drug susceptibility are still an understudied field. Here, we show that the clinically relevant drug fosfomycin (FOS) penetrates through the OM of *E. coli* via porins OmpF, OmpC, and LamB. In the absence of these porins, the susceptibility of FOS is clearly reduced. One very important conclusion from our study is that the role of these porins in FOS uptake and antibacterial action can only be appreciated and thus is only physiologically and clinically relevant when rapid IM uptake and fast depletion from the periplasm is assured (i.e., in the presence of G6P).

## Methods

### Bacterial Strains and Chemicals

In this study, we used *E. coli* BW25113 (wt) and the respective *ompC*, *ompF*, and *lamB* porin mutant strains. A list of all strains used in this study can be found in the Supporting Information, Table S2. In all experiments, the pH of the lysogeny broth medium (LB Lennox, Carl Roth, Karlsruhe, Germany) was adjusted to 7.4 prior to autoclaving. *E. coli* cultures were routinely started by inoculating 10 mL of LB medium in a 100 mL flask with a single colony, and the bacteria were grown at 37 °C and 130 rpm for 20 h. Glucose 6-phosphate dipotassium salt (G6P) and fosfomycin (FOS) disodium salt were purchased from Sigma-Aldrich (Darmstadt, Germany). MIC Test Strips (MTS) for FOS (0.064–1024 μg/mL with 50 μg/mL G6P) and for meropenem (MRP, 0.002–32 μg/mL) were obtained from Liofilchem (Roseto degli Abruzzi, Italy).

### Porin Deletion Mutants

*E. coli* BW25113 Δ*ompF*, Δ*ompC*, and Δ*ompF*Δ*ompC* markerless deletion mutants were previously generated.^45^*E. coli* BW25113 Δ*lamB::kan* strain was from the Keio collection.^60^*E. coli* BW25113 Δ*ompF*Δ*ompC*Δ*lamB* was constructed from the Δ*ompF::kan* strain of the Keio collection,^60^ in which the kanamycin cassette was excised with the pCP20 plasmid according to a published protocol.^61^ For the subsequent deletions of *ompC* and *lamB* genes in the Δ*ompF* markerless mutant, primers were used that contained sequences for amplification of the kanamycin resistance cassette of the pKD13 plasmid that were flanked by a homologous region of the target genes, *ompC* or *lamB* (see Table S1). *E. coli* Δ*ompF* cells were transformed with the pKD46 plasmid and subsequently with the obtained PCR products. Homologous recombination was initiated by λ Red recombinase from the pKD46 plasmid. Successful integration of the kanamycin cassette was controlled by cell growth on LB agar plates, supplemented with 50 μg/mL kanamycin. The kanamycin cassette was finally removed with the pCP20 plasmid.^61^ Correct deletion of the *ompF*, *ompC*, and *lamB* genes was confirmed by PCR and Sanger sequencing.

### FOS Susceptibility by Agar Dilution

MIC values by agar dilution method were determined following the guidelines of the Clinical and Laboratory Standard Institute;^62^ however, LB agar instead of Müller-Hinton agar plates were used. *E. coli* strains were grown in LB medium for 20 h and bacteria were diluted in 0.85% NaCl to 0.5 McFarland turbidity standard, corresponding to ∼10^8^ cfu/mL.^63^ Bacteria were further diluted 1:10 in 0.85% NaCl and 2 μL of this dilution (∼2 × 10^4^ cfu) was spotted on LB pH 7.4 with 1.5% agar plates containing 2-fold dilutions of FOS at a final concentration of 0.25 to 512 μg/mL or no FOS (control) in the absence or presence of 25 μg/mL G6P. Agar plates were then incubated at 37 °C and after 20 h the MIC values were determined as the lowest antibiotic concentration at which no growth was observed, ignoring the emergence of a single resistant *E. coli* colony on the agar plate (Figure S2), according to^64^ and following the CLSI recommendations.^62^

### Determination of FOS and Meropenem Susceptibility by Gradient Strip Test

MIC values by gradient strip test were determined according to the manufacturer’s recommendations, using LB agar plates.^41^ Briefly, *E. coli* cells were grown overnight and diluted in 0.85% NaCl to a 0.5 McFarland turbidity standard. Bacteria were distributed with a sterile cotton swab (Carl Roth GmbH + Co.KG, Karlsruhe, Germany) on the LB plus 1.5% agar plates (17.5 mL, pH 7.4), MIC Test Strips were placed on the inoculated plates, which were finally incubated for 20 h at 37 °C. MIC for FOS and meropenem was determined as the lowest antibiotic concentration for which no bacterial growth was detected. The presence of resistant colonies in the zone of inhibition is characteristic of the antibiotic FOS, and these colonies were not considered during the final MIC evaluation.^41^

### FOS Susceptibility in Liquid Medium

Growth of *E. coli* BW25113 wt and porin mutant strains with FOS was also investigated in liquid LB medium. Bacteria were grown in a 24-well plate (Greiner bioone Cellstar, flat bottom for cell culture) for 20 h at 37 °C under shaking at 365 rpm in a final volume of 500 μL per well. Optical density at 600 nm (OD_600_) was measured every 5 or 10 min in a microplate spectrophotometer BioTek Epoch 2 (Agilent, Santa Clara).

Overnight cultures of *E. coli* were diluted to a final OD_600_ of 0.05 (corresponds to OD_600_ of 0.09 in the microplate reader) in LB medium with 25 μg/mL G6P and grown until an OD_600_ of 1.96 was registered (corresponding to OD_600_ of 0.3 in the microplate reader). At this point, cells were treated with FOS at the final concentrations of 0, 1, 2, 4, and 8 μg/mL, and OD_600_ was further monitored for 20 h. Row Excel data were extracted from the BioTek Epoch 2 reader and visualized using the program GraphPad Prism 8.4.3.

### Quantification of Glucose 6P in Culture Supernatant by HPLC-MS

Overnight cultures of *E. coli* BW25113 parental strain and Δ*ompF*Δ*ompC* and Δ*ompF*Δ*ompC*Δ*lamB* mutants were diluted in LB medium supplemented with 25 μg/mL G6P to a final OD_600_ of 0.05, which was determined in a cuvette. The bacterial growth was then followed in the Epoch 2 microplate reader as previously described and samples were collected at OD_600_ of 0.09 (time point 0), 0.1, 0.15, 0.2, 0.25, and 0.3, corresponding to OD_600_ of 0.05, 0.074, 0.54, 1.02, 1.49, and 1.96 in the cuvette, respectively. Bacterial cultures were centrifuged at 13,500 rpm for 10 min, and 200 μL of the supernatant was collected and stored at 4 °C. To ensure quantification, 25 μg/mL G6P was diluted with serial dilutions with a factor of 1.25 in LB culture medium. The G6P samples of the standard curve and the control sample (LB medium) were also centrifuged and 200 μL of each were stored at 4 °C. All samples were finally dried in a SpeedVac vacuum centrifuge (Martin Christ Gefriertrocknungsanlagen GmbH, Osterode am Harz, Germany) for 4 h at 30 °C, and the pellet was resuspended in 20 μL of ddH_2_O.

G6P amounts in the culture supernatant were quantified with an electrospray ionization-time-of-flight (ESI-TOF) mass spectrometer (MicrO-TOF II, Bruker) coupled to the UltiMate 3000 high-performance liquid chromatography (HPLC) system (Dionex, Sunnyvale). 3 μL of each sample was injected onto a SeQuant ZIC-pHILIC column (PEEK 150 × 2.1 mm, 5 μm, Merck, Darmstadt, Germany) and analyzed at 30 °C in a mass range from 85 to 900 *m*/*z*. Samples were separated at a flow rate of 0.2 mL/min using a 35 min gradient program^65^ with small modifications: 5 min of 82% buffer A (CH_3_CN) and 18% buffer B (100 mM (NH_4_)_2_CO_3_, pH 8.9) were followed by a 20 min linear gradient to 42% buffer A and by an equilibration step of 10 min at 82% buffer A. Extracted ion chromatograms (EIC) and the relative areas under the curve (AUCs) for G6P (observed [M – H]^−^ = 259.022 *m*/*z*, maximal peak intensity at 17 min) were calculated using the UmetaFlow program,^66^ with a mass tolerance of 0.02 Da.

### RNA Extraction

*E. coli* BW25113 wt, Δ*ompF*Δ*ompC*, and Δ*ompF*Δ*ompC*Δ*lamB* mutant strains were grown in the microplate reader in LB medium with 25 μg/mL G6P to a final OD_600_ to 0.3, as described in “FOS susceptibility in liquid medium”. After centrifugation (13.500 rpm, 10 min), the bacterial pellet was frozen immediately at −80 °C. The cellular RNA was extracted using the *Quick-*RNA Fungal/Bacterial Miniprep kit (Zymo Research, Freiburg, Germany), according to the manufacturer’s instructions, and finally eluted in 50 μL of DNase/RNase-free water. The concentration and purity of the samples were measured with the Nanodrop (NanoPhotometer N60, Implen, Munich, Germany) and a DNase I reaction (RNase-free, Thermo Fisher Scientific, Waltham) was performed to remove residual genomic DNA. Briefly, each RNA sample (49 μL, containing maximal 8 μg/mL total RNA) was incubated with 6 μL of incubation buffer (10×), 0.8 μL of enzyme, and DNase/RNase-free water in a final volume of 60 μL. The samples were then incubated at 37 °C for 20 min, 2 μL of 0.5 M ethylenediaminetetraacetic acid (EDTA) (pH 0.8, Invitrogen by Thermo Fisher Scientific, Waltham) were added and the reaction was stopped by incubation at 75 °C for 10 min. The RNA samples were then cleaned from EDTA and concentrated using the RNA Clean and Concentrator Kit-5 (Zymo Research, Freiburg, Germany) according to the manufacturer’s instructions. The concentration and purity of the RNA samples were determined by NanoDrop, all samples were diluted to a final concentration of 80 ng/μL and were frozen at −80 °C.

### Analysis of *uhpT* Expression by RT-qPCR

*uhpT* expression in the BW25113 wt, Δ*ompF*Δ*ompC*, and Δ*ompF*Δ*ompC*Δ*lamB* strains was analyzed by RT-qPCR. Therefore, specific primers for the *uhpT* and the reference gene 16S rRNA (BW25113_RS19980) were designed (Merck, Darmstadt, Germany) and are listed in Table S1. For each primer pair, biological replicates were analyzed by RT-qPCR in technical duplicates. RNA samples were diluted to a concentration of 8 ng/μL in DNase-RNase-free water, and the QuantiFast SYBR Green RT-PCR Kit (QIAGEN, Hilden, Germany) was used to run the RT-qPCR in a 96-well plate (MicroAmp Optical 96-Well Reaction Plate, Applied Biosystems by Thermo Fisher Scientific, Waltham). Briefly, for each sample, a 10 μL reaction mix was prepared, containing 5 μL of 2× QuantiFast SYBR Green RT-PCR Master Mix, 0.1 μL of QuantFast RT Mix enzyme, 1 μL of 10 μM forward and reverse primers, 1 μL of RNA sample (8 ng/μL), and 1.9 μL of water. The experiment was run in a QuantStudio3 thermocycler (applied biosystems by Thermo Fisher Scientific, Waltham) and data were analyzed by the Design and Analysis software 2.6.0 (Thermo Fisher Scientific). The relative *uhpT* expression was calculated as 2^–ΔΔCt^ and data were presented in GraphPad Prism 8.4.3.

### Electrophysiological Zero-Current Assays

The recombinant porins OmpF, OmpC, LamB, and PhoE were isolated as previously described,^67^ using pG expression vectors.^68,69^ Planar lipid bilayer and electrical recording multichannel measurements were performed as described.^43,58^ Briefly, an aperture in a Teflon septum with a diameter of 100–120 μm was prepainted with hexadecane dissolved in *n*-hexane at 1% (v/v) and dried for 10–15 min to evaporate the solvent. Bilayers were made with 1,2-diphytanoyl-*sn*-glycero-phosphatidyl-choline (Avanti Polar Lipid, Alabaster, AL) at a concentration of 5 mg/mL in *n*-pentane. Stock solutions (0.5 mg/mL) of the outer-membrane porins OmpF, OmpC, LamB, and PhoE were diluted 10^3^- to 10^5^-fold using Genapol X-080 (1% v/v). A small volume from the dilution was added to the electric ground side of the chamber containing 2.5 mL of 25 mM FOS for single-channel measurements. For reversal potential measurements, the gradient was increased on the ground side to 90 mM FOS. Throughout the experiments, the pH was maintained using a HEPES buffer at 7.0. We used a 200B Axopatch with the company software Clampfit (Molecular Device). Standard Ag/AgCl electrodes (Metrohm) were used to detect the ionic current in single-channel experiments. To estimate the flux we followed previous calculations.^43,58^ The Goldman–Hodgkin–Katz (GHK) equation gave an estimate for the permeability ratio between the FOS/sodium flux. We extrapolated the single-channel conductance to 1 μM (about MIC values) and used the flux ratio to estimate the number of FOS molecules per monomer channel and per second.

### Transcriptomics Analysis

Overnight cultures of *E. coli* BW25113 were diluted in LB medium supplemented with 25 μg/mL G6P to a final OD_600_ of 0.05 (corresponding to an OD_600_ of 0.09 in the Epoch2 microplate reader), and growth was continued as previously described until an OD_600_ of 1.9 was reached (OD_600_ = 0.3 in the Epoch 2). Samples of three biological replicates were collected and centrifuged (13.500 rpm, 10 min), and the pellet was frozen at −80 °C. The cellular RNA was extracted as previously described, and the quality and concentration of each sample were assessed by Nanodrop.

For library preparation, Illumina Stranded Total RNA Prep Ligation with Ribo-Zero Plus was used according to manufacturer’s instructions together with IDT for Illumina DNA/RNA UD Indexes Set A. The input amount was 300 ng of RNA in 11 μL; QC measurements were performed using Qubit (Qubit RNA BR Assay Kit, Qubit dsDNA HS Assay Kit) and Bioanalyzer (Bioanalyzer High sensitivity DNA Kit).

Sequencing was run on a NextSeq 500 (1 × 75 bp single-end reads).

Sequencing statistics including the quality per base and adapter content assessment of the resulting transcriptome sequencing data were conducted with FastQC v0.11.8 [https://www.bioinformatics.babraham.ac.uk/projects/fastqc/]. All read mappings were performed against the strain *E. coli* BW25113 (RefSeq ID: NZ_CP009273.1). The mappings of all samples were conducted with HISAT2 v2.1.0.^70^ As parameters, spliced alignment of reads was disabled and strand-specific information was set to reverse-complement (HISAT2 parameter --no-spliced-alignment and --rna-strandness “R”). The resulting mapping files in SAM format were converted to BAM format using SAMtools v1.9.^71^ Mapping statistics, including strand specificity estimation and percentage of mapped reads, were conducted with the RNA-Seq module of QualiMap2 v2.2.2-a.^72^ Gene counts for all samples were computed with featureCounts v1.6.4,^73^ where the selected feature type was set to transcript records (featureCounts parameter -t transcript). A quality check for ribosomal rRNA was performed with a self-written script based on the absolute counts of annotated rRNAs. The reads counts were calculated using a TPM (transcript per million) normalization.^74^

All RNA-Seq Illumina read files as well as the raw gene counts have been deposited in NCBI’s Gene Expression Omnibus and are accessible under accession number GSE236554.

## Supporting Information Available

Verification of *ompC*, *ompF*, and *lamB* gene deletions by colony PCR (Figure S1); *E. coli* wild type and porin mutant strains susceptibility to fosfomycin by agar dilution method (Figure S2); comparison of fosfomycin and meropenem sensitivity of wt and porin mutant strains by gradient strip test (Figure S3); comparison of *E. coli* wt and porin mutant strains susceptibility to FOS by gradient strip test ± LamB induction (Figure S4); HPLC-MS analysis of G6P in *E. coli* cultures grown in liquid LB (Figure S5); transcripts per million for *E. coli* BW25113 genes encoding porins and the inner-membrane fosfomycin transporters UhpT and GlpT (Figure S6); and primers and strains used in this study (Tables S1 and S3) (PDF)
