# Supplementary material for: Fosfomycin Uptake in Escherichia coli Is Mediated by the Outer-Membrane Porins OmpF, OmpC, and LamB
Source: ACS Infect Dis. 2023 Dec 17;10(1):127–37. doi: 10.1021/acsinfecdis.3c00367 (PMC10789261; doi:10.1021/acsinfecdis.3c00367)
Supplement: Supplementary file 1 — id3c00367_si_001.pdf [file id3c00367_si_001.pdf]

Supporting information

Fosfomycin uptake in *Escherichia coli* is mediated by the outer membrane porins OmpF, OmpC and LamB

[1]Martina Bianchi, [2]Mathias Winterhalter, [3]Theresa Anisja Harbig, [4]Daniel Hörömpöli, [2]Ishan Ghai, [3,5]Kay Nieselt, [4,5]Heike Brötz-Oesterhelt, [1,5]Christoph Mayer* and [1,5]Marina Borisova-Mayer*

[1]Department of Organismic Interactions, Interfaculty Institute of Microbiology and Infection Medicine (IMIT), University of Tübingen, 72076, Tübingen, Germany

[2]Department of Life Sciences and Chemistry, Constructor University, 28759, Bremen, Germany

[3]Institute for Bioinformatics and Medical Informatics, University of Tübingen, 72076, Tübingen, Germany

[4]Department of Microbial Bioactive Compounds, IMIT, University of Tübingen, 72076, Tübingen, Germany

[5]Cluster of Excellence "Controlling Microbes to Fight Infections" University of Tübingen, 72076, Tübingen, Germany

*christoph.mayer@uni-tuebingen.de

*marina.borisova@uni-tuebingen.de

Methods

Colony PCR

Deletion of the *ompC*, *ompF* and *lamB* genes in *E. coli* BW25113 parental and porin mutant strains was verified by colony PCR. Therefore, a Taq DNA Polymerase Master Mix Red (GENAXXON bioscience, Ulm, Germany) was used according to the instructions provided by the company to amplify the porin genes. Primers, used to perform the colony PCR are listed in table S1. After the PCR reaction, DNA products were loaded on 1% agarose gels and visualized by UV light with MIDORI Green advance dye (NIPPON Genetics EUROPE).

Supplementary figures

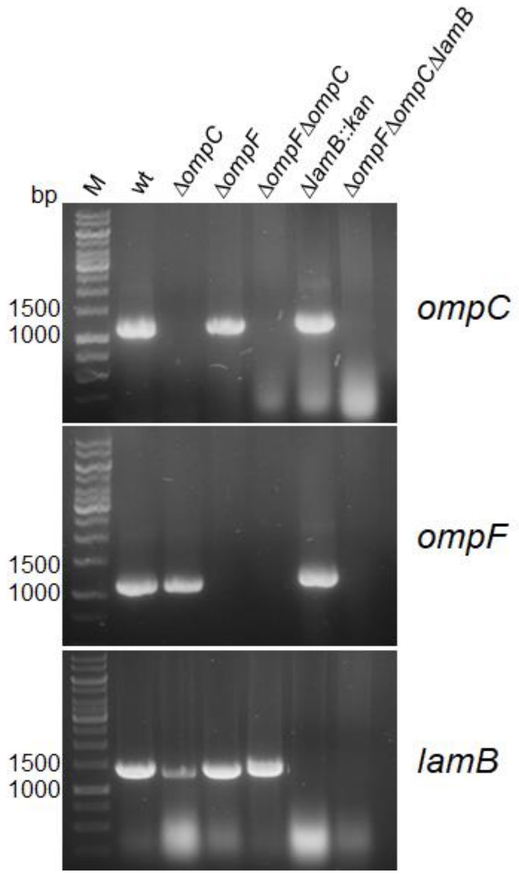

Figure S1. Verification of *ompC*, *ompF* and *lamB* gene deletions by colony PCR. *E. coli* BW25113 wild type (wt), Δ*ompC*, Δ*ompF*, Δ*ompF*Δ*ompC*, Δ*lamB::kan* and Δ*ompF*Δ*ompC*Δ*lamB* strains were analyzed by colony PCR with specific primers, amplifying the *ompC*, *ompF* and *lamB* genes. M, 1 kB DNA ladder; expected size of the PCR products for *ompC* (1128 bp), for *ompF* (1113 bp) and *lamB* (1321 bp).

A.

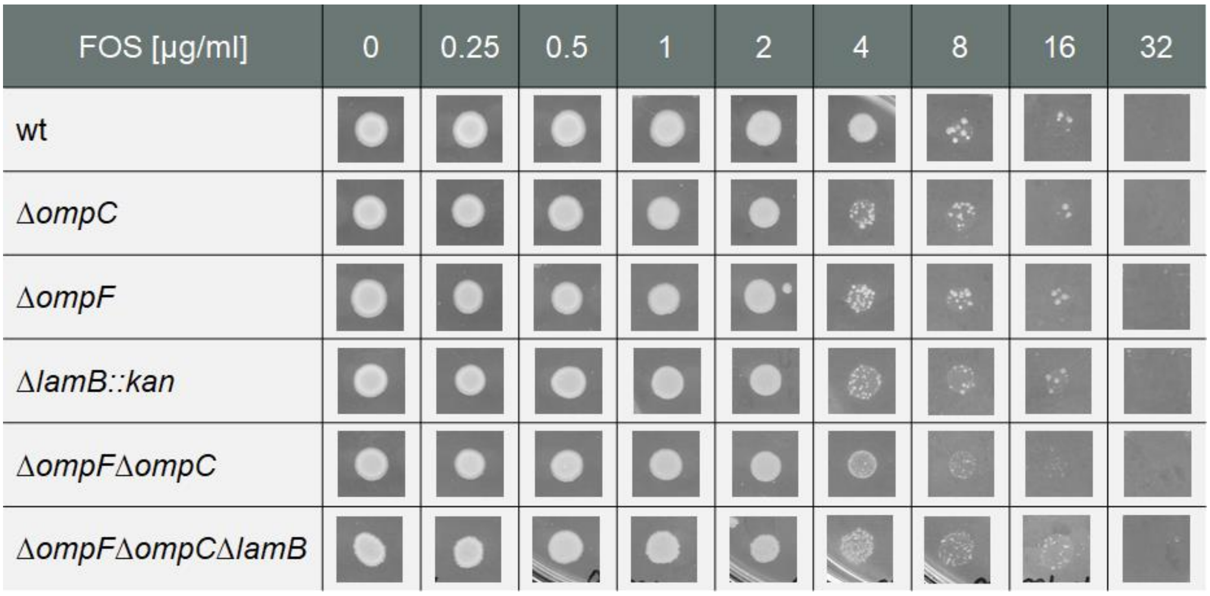

B.

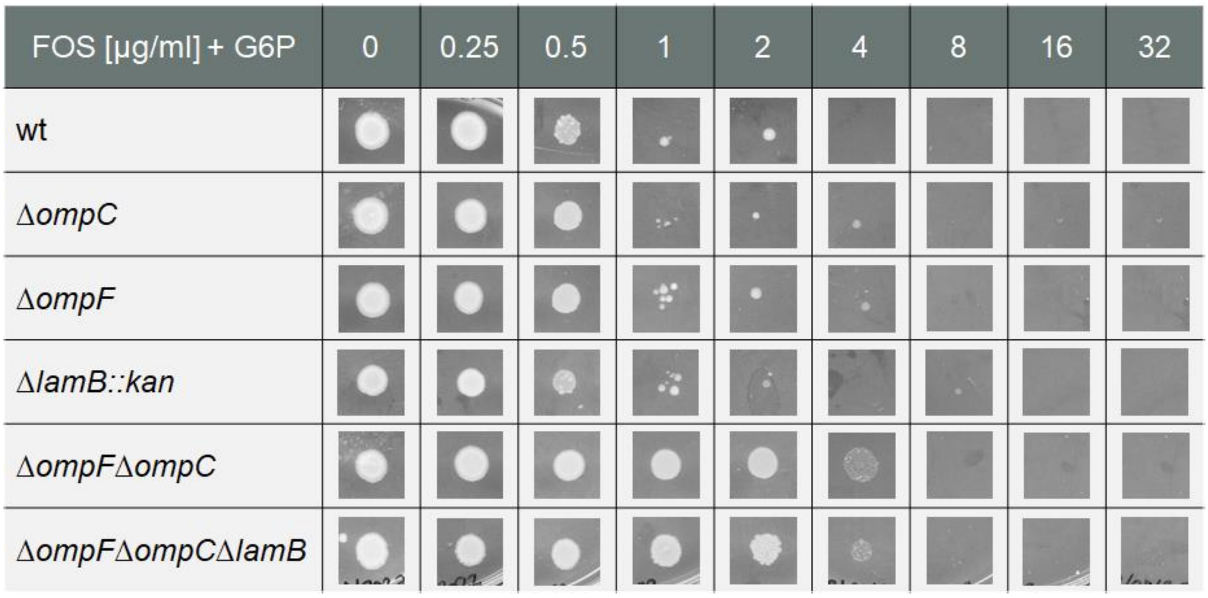

Figure S2. *E. coli* wild type and porin mutant strains susceptibility to fosfomycin by agar dilution method. Overnight cultures of *E. coli* BW25113 wt, *ΔompC*, *ΔompF*, *ΔlamB::kan* single mutants, as well as *ΔompFΔompC* and *ΔompFΔompCΔlamB* mutants were diluted in 0.85% NaCl and 2 x 10^4 cfu were spotted on LB agar plates containing 0.25 to 32 µg/ml gradient dilutions of fosfomycin (FOS) or only LB plates (control, 0) in the **A.** absence or **B.** presence of 25 µg/ml of G6P for UhpT expression. MIC for FOS was determined as the lowest concentration for which no growth was detectable, ignoring the occurrence of a single resistant colony. Representative pictures of the agar dilution experiment performed in three biological replicates, each done in two technical replicates.

A. Fosfomycin (FOS)

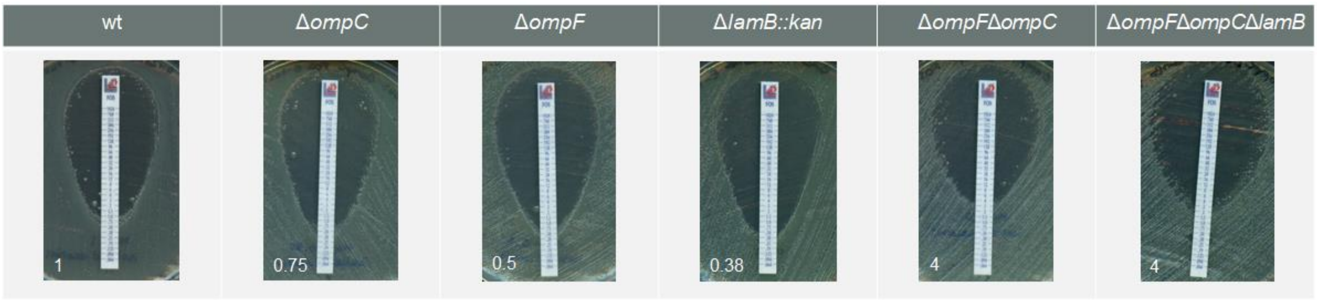

B. Meropenem

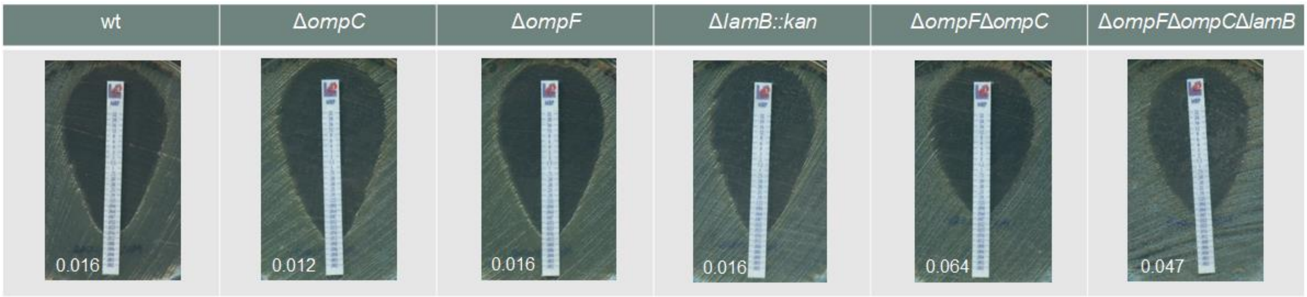

C.

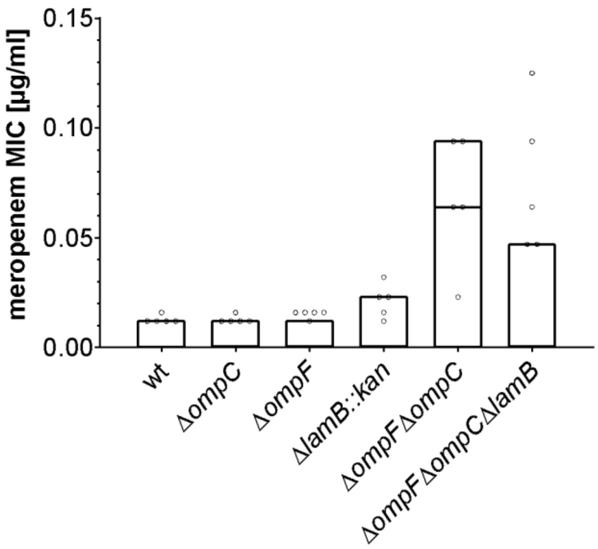

Figure S3. Comparison of fosfomycin and meropenem sensitivity of wt and porin mutant strains by gradient strip test . Overnight cultures of *E. coli* wt, *ΔompC*, *ΔompF, lamB::kan*, *ΔompFΔompC* and *ΔompFΔompCΔlamB* mutants were diluted in 0.85% NaCl to McFarland 0.5. E-test susceptibility tests for **A.** Fosfomycin (FOS) and **B.** for meropenem (MRP) were assayed on LB agar plates with MIC Test Strips (MTS^{TM}) with 50 µg/ml G6P . Determined MIC values [µg/mL] are shown as numbers on the lower left corner. **C.** MICs for meropenem are presented as mode values of five biological replicates.

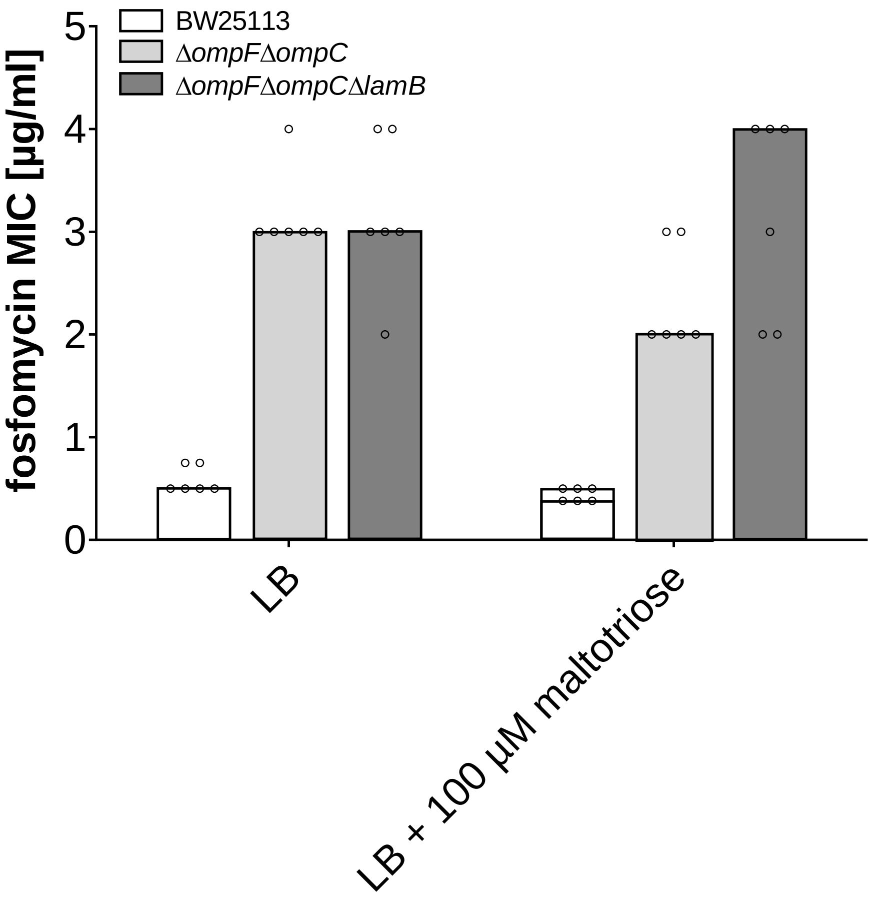

Figure S4. Comparison of *E. coli* wt and porin mutant strains susceptibility to FOS by gradient strip test ± LamB induction. Overnight cultures of *E. coli* wt, Δ*ompF*Δ*ompC* and Δ*ompF*Δ*ompC*Δ*lamB* strains were diluted in 0.85% NaCl to McFarland 0.5. MIC gradient strips were applied to assess the FOS susceptibility of all strains on LB agar plates containing 0 or 100 µM maltotriose. The MICs [µg/ml] from three biological replicates, each done in technical duplicates, are presented as mode values. *E. coli* wt strain, which was grown with maltotriose has two mode values of 0.5 and 0.38 µg/ml.

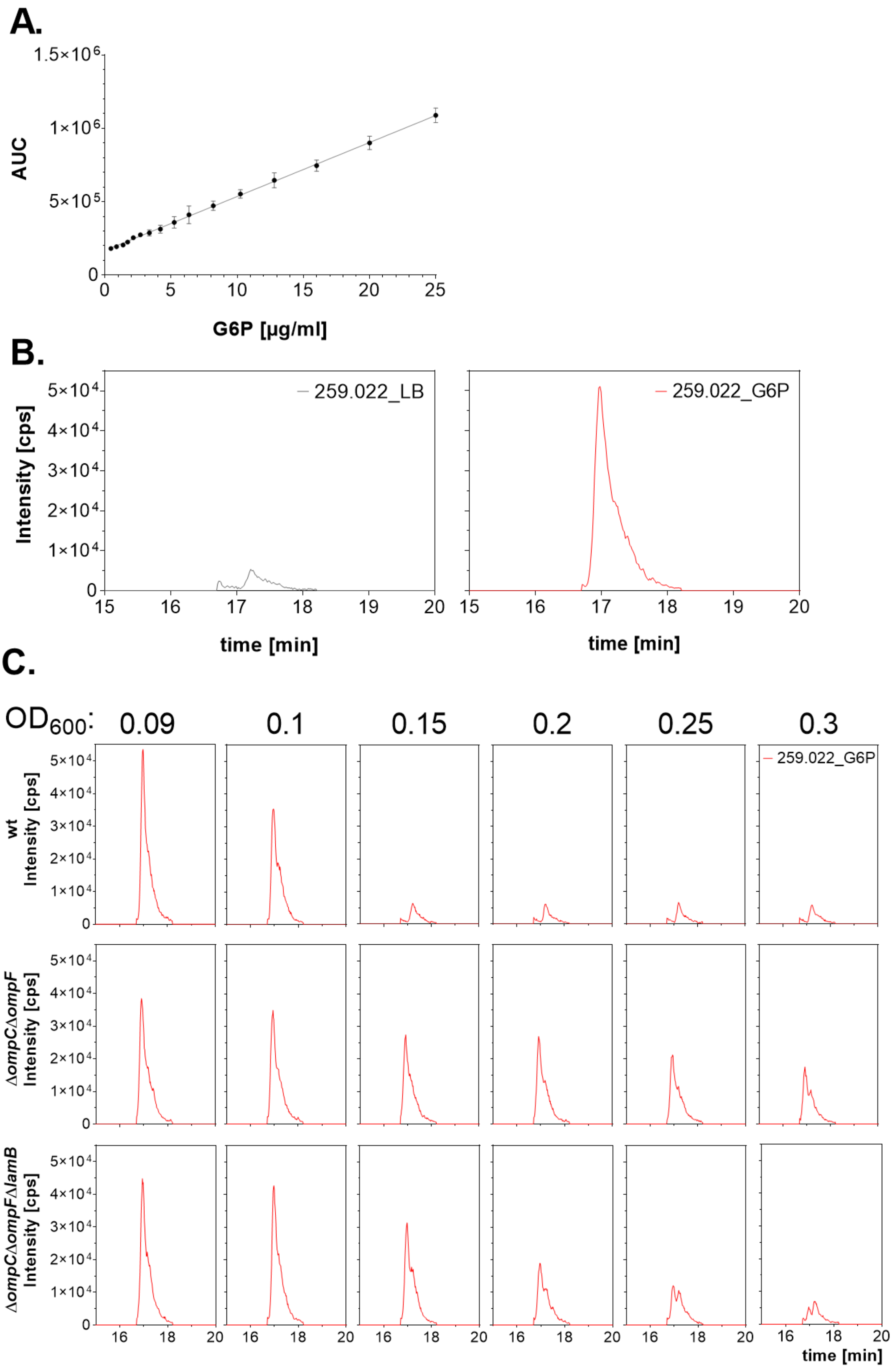

A.
AUC
1.5×10^6
1×10^6
5×10^5
0
0 5 10 15 20 25
G6P [µg/ml]
B.
Intensity [cps]
5×10^4
4×10^4
3×10^4
2×10^4
1×10^4
0
259.022_LB
259.022_G6P
15 16 17 18 19 20
time [min]
C.
OD600: 0.09 0.1 0.15 0.2 0.25 0.3
259.022_G6P
wt
Intensity [cps]
ΔompCΔompF
Intensity [cps]
ΔompCΔompFΔlamB
Intensity [cps]
16 18 20
time [min]

Figure S5. HPLC-MS analysis of G6P in *E. coli* cultures grown in liquid LB. *E. coli* wt, Δ*ompF*Δ*ompC* and Δ*ompF*Δ*ompC*Δ*lamB* samples were collected while growing in liquid LB in the microplate reader, whenever an OD_{600} increase of 0.05 was registered. The supernatant was then analyzed by HPLC-MS. G6P could be identified with an observed mass $[M-H]^-$ of 259.022 m/z and maximal peak intensity at 17 min. Extracted ion chromatograms (EICs) and the relative area under the curve (AUC) (baseline = 50 cps) were obtained through the UMetaFlow program and the results were plotted in GraphPad Prism 8.4.2. **A.** A standard curve of G6P was created by diluting an initial amount of 25 µg/ml further in LB (dilution factor of 1.25). The experiment was performed in triplicates and mean AUCs values ± SEM are represented. **B.** Representative EICs. On the left, a background signal given by a metabolite with the same m/z found in LB without G6P addition. On the right, representative EIC of G6P in LB, 25 µg/ml. **C.** Representative EICs of G6P detected in the supernatant of each sample.

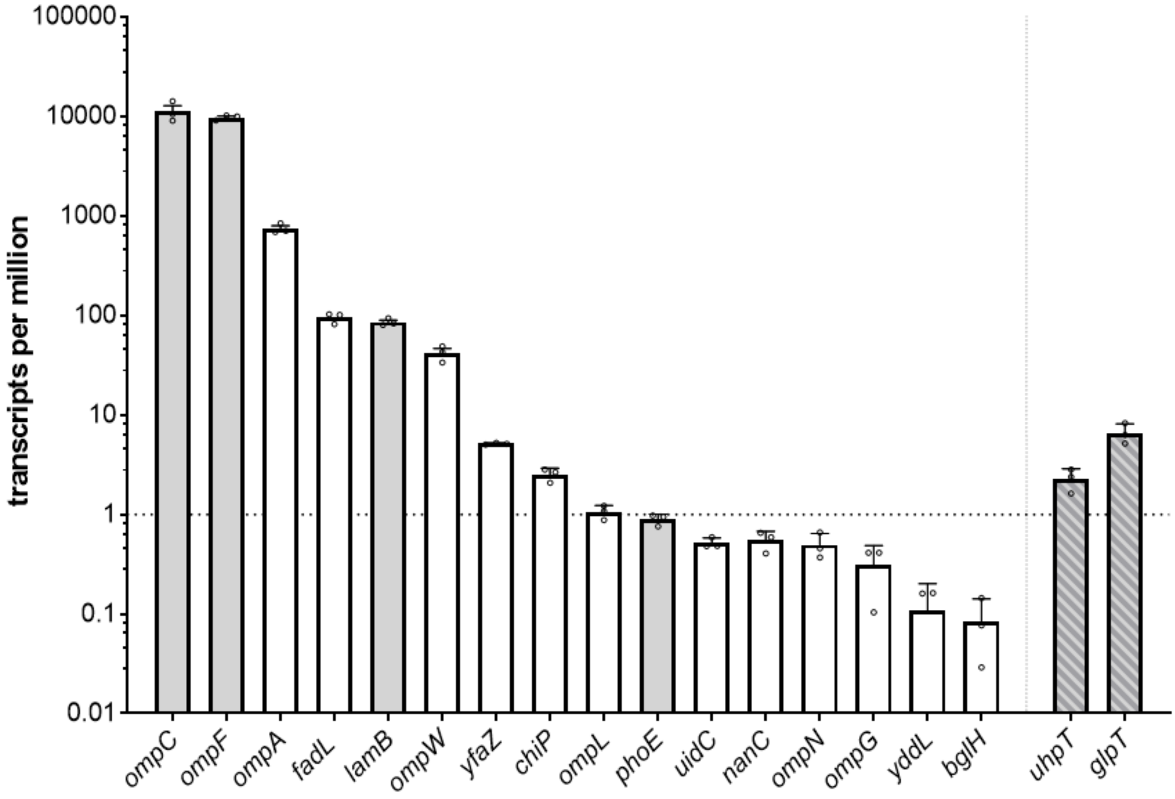

Figure S6. Transcripts per million for *E. coli* BW25113 genes encoding porins and the inner membrane fosfomycin transporters UhpT and GlpT.

E. coli BW25113 cells were grown in LB medium until an OD_{600} of 0.3 in the microplate reader, corresponding to OD_{600} 1.9 in the cuvette, was reached. Cells were harvested, total RNA was extracted, and after Illumina RNA-sequencing, reads were mapped and normalized as transcripts per million (TPM), here presented in a log-10 scale (see Y-axis). The experiment was performed in three biological replicates and data are presented as mean + SEM.

Supplementary Tables

Table S1. Primers used in this study.

**Name**	**Nucleotide sequence (5´to 3´)**	**Application**
MB6_BW25113.ompC.FW	TAAGCAGCTAGCATGAAAGTTAAAGTACTGTCCCTCCTG	Colony PCR, amplification of *ompC*
MB7_BW25113.ompC.REV	TAAGCAAAGCTTTTAGAACTGGTAAACCAGACCCAG	Colony PCR, amplification of *ompC*
MB8_BW25113.ompF.FW	TAAGCAGCTAGCATGATGAAGCGCAATATTCTGGCA	Colony PCR, amplification of *ompF*
MB9_BW25113.ompF.REV	TAAGCAAAGCTTTTAGAACTGGTAAACGATACCCACAGC	Colony PCR, amplification of *ompF*
MB10_BW25113.lamB.FW	GCAAACTTCCTCTGGCGGTTG	Colony PCR, amplification of *lamB*
MB11_BW25113.lamB.REV	CACCAGATTTCCATCTGGGCACC	Colony PCR, amplification of *lamB*
ompC-KO_for	TGTTCGATATCAATCGAGATTAGAACTGGTAAACCAGACCATTCCGGGGATCCGTCGACC	*ompC* deletion to generate Δ*ompF*Δ*ompC*Δ*lamB*
ompC-KO_rev	AAAAGCAAATAAAGGCATATAACAGAGGGTTAATAACATGTGTAGGCTGGAGCTGCTTCG	*ompC* deletion to generate Δ*ompF*Δ*ompC*Δ*lamB*
KO-lamB_for	GATGTGAAAAAAGAAAAGCAATGACTCAGGAGATAGAATGATTCCGGGGATCCGTCGACC	*lamB* deletion to generate Δ*ompF*Δ*ompC*Δ*lamB*
KO-lamB_rev	TCCGGCCCAGGTTTTGCTATTACCACCAGATTTCCATCTGTGTAGGCTGGAGCTGCTTCG	*lamB* gene deletion to generate Δ*ompF*Δ*ompC*Δ*lamB*
MB12_BW25113.16SPCR.FW	GGGTTGTAAAGTACTTTCAGCGGG	16S rRNA by RT-qPCR (reference gene)
MB13_BW25113.16SPCR.REV	TGACTTAACAAACCGCCTGCG	16S rRNA by RT-qPCR (reference gene)
MB14_BW25113.uhpTPCR.FW	TGAAACTCTCTAAAGCGGTGGCG	*uhpT* by RT-qPCR
MB14_BW25113.uhpTPCR.REV	GCATGTTGATACACACCGAGCG	*uhpT* by RT-qPCR

Table S2. Strains, used in this study.

Strain	Genotype/characteristics	Source
E. coli BW25113	Wild type; F-, Δ*(araD-araB)567,* Δ*lacZ4787(::rrnB-3)*, *λ-, rph-1,* Δ*(rhaD-rhaB)568, hsdR514*	[2]
E. coli BW25113 Δ*ompC*	F-, Δ*(araD-araB)567*, Δ*lacZ4787(::rrnB-3), λ-*, Δ*ompC768, rph-1,* Δ*(rhaD-rhaB)568, hsdR514*	[3]
E. coli BW25113 Δ*ompF*	F-, Δ*(araD-araB)567*, Δ*lacZ4787(::rrnB-3)*, λ-, Δ*ompF746, rph-1,* Δ*(rhaD-rhaB)568*, *hsdR514*	[3]
E. coli BW25113 *lamB::kan*	F-, Δ*(araD-araB)567,* Δ*lacZ4787(::rrnB-3), λ-,* Δ*lamB732::kan, rph-1*, Δ*(rhaD-rhaB)568*, *hsdR514*	[4,2]
E. coli BW25113 *ΔompFΔompC*	F-, Δ*(araD-araB)567,* Δ*lacZ4787(::rrnB-3), λ-,* Δ*ompF746,* Δ*ompC768, rph-1,* Δ*(rhaD-rhaB)568, hsdR514*	[3]
E. coli BW25113 *ΔompFΔompCΔlamB*	F-, Δ*(araD-araB)567,* Δ*lacZ4787(::rrnB-3), λ-*, Δ*lamB732,* Δ*ompF746*, Δ*ompC768, rph-1,* Δ*(rhaD-rhaB)568*, *hsdR514*	this study

References

1. Xu, C.; Lin, X.; Ren, H.; Zhang, Y.; Wang, S.; Peng, X., Analysis of outer membrane proteome of *Escherichia coli* related to resistance to ampicillin and tetracycline. *Proteomics* **2006,** *6* (2), 462-73.
2. Baba, T.; Ara, T.; Hasegawa, M.; Takai, Y.; Okumura, Y.; Baba, M.; Datsenko, K. A.; Tomita, M.; Wanner, B. L.; Mori, H., Construction of *Escherichia coli* K-12 in-frame, single-gene knockout mutants: the Keio collection. *Mol Syst Biol* **2006,** *2*, 2006 0008.
3. Bafna, J. A.; Sans-Serramitjana, E.; Acosta-Gutiérrez, S.; Bodrenko, I. V.; Hörömpöli, D.; Berscheid, A.; Brötz-Oesterhelt, H.; Winterhalter, M.; Ceccarelli, M., Kanamycin uptake into *Escherichia coli* is facilitated by OmpF and OmpC porin channels located in the outer membrane. *ACS Infect Dis* **2020,** *6* (7), 1855-1865.
4. Datsenko, K. A.; Wanner, B. L., One-step inactivation of chromosomal genes in *Escherichia coli* K-12 using PCR products. *Proc Natl Acad Sci U S A* **2000,** *97* (12), 6640-5.
